# Acoustofluidics-Assisted Coating of Microparticles

**DOI:** 10.3390/polym15194033

**Published:** 2023-10-09

**Authors:** Ming-Lin Yeh, Geng-Ming Chang, Yi-Je Juang

**Affiliations:** 1 Department of Chemical Engineering, National Cheng Kung University, No. 1 University Road, Tainan 70101, Taiwan; 2 Core Facility Center, National Cheng Kung University, No. 1 University Road, Tainan 70101, Taiwan; 3 Research Center for Energy Technology and Strategy, National Cheng Kung University, No. 1 University Road, Tainan 70101, Taiwan

**Keywords:** microfluidics, traveling surface acoustic wave, conformal coating, polyelectrolyte, polydimethylsiloxane

## Abstract

Microparticles have been applied in many areas, ranging from drug delivery, diagnostics, cosmetics, personal care, and the food industry to chemical and catalytic reactions, sensing, and environmental remediation. Coating further provides additional functionality to the microparticles, such as controlled release, surface modification, bio-fouling resistance, stability, protection, etc. In this study, the conformal coating of microparticles with a positively charged polyelectrolyte (polyallylamine hydrochloride, PAH) by utilizing an acoustofluidic microchip was proposed and demonstrated. The multiple laminar streams, including the PAH solution, were formed inside the microchannel, and, under the traveling surface acoustic wave, the microparticles traversed through the streams, where they were coated with PAH. The results showed that the coating of microparticles can be achieved in a rapid fashion via a microfluidic approach compared to that obtained by the batch method. Moreover, the zeta potentials of the microparticles coated via the microfluidic approach were more uniform. For the unfunctionalized microparticles, the charge reversal occurred after coating, and the zeta potential increased as the width of the microchannel or the concentration of the PAH solution increased. As for the carboxylate-conjugated microparticles, the charge reversal again occurred after coating; however, the magnitudes of the zeta potentials were similar when using the microchannels with different widths or different concentrations of PAH solution.

## 1. Introduction

Microparticles have been utilized in many areas such as drug delivery [1], diagnostics [2], cosmetics, personal care [3], the food industry [4], chemical and catalytic reactions [5], sensing [6], and environmental remediation [7]. Coating further provides additional functionality to the microparticles such as controlled release, surface modification, bio-fouling resistance, stability, protection, etc. Compared to conventional coating methods, which are usually labor-intensive, complex, and have the potential to cause issues of sample contamination, microfluidics-assisted coating provides a simple and easy alternative that could circumvent the problems of contaminating the sample. Various strategies have been exploited. For example, Tsai et al. applied a magnetic force to move the magnetic particles across the water–oil interface inside the microchannel, where the particles were coated with a thin film of water [8]. A conformal coating of non-spherical magnetic particles was also attempted and demonstrated [9]. Tarn et al. functionalized living cells with magnetic nanoparticles and utilized a magnetic force to deflect the cells across the polyelectrolyte streams, resulting in coating the cells with a polyelectrolyte [10]. Hemptinne et al. constructed a co-flow microfluidic chips with three parallel liquid streams, where diffusion between the streams was hindered by the channel walls. The magnetic particles then moved across the streams through the openings due to the magnetic force [11]. As the particles moved downstream, they alternated their flow path between streams, and multiple coatings were achieved. In their study, the overall processing time for multiple coatings was reduced from 30 to 45 s using a bulk layer-by-layer method to 3 s. Alorabi et al. coated oil droplets containing magnetic nanoparticles with polyelectrolytes by moving them across the laminar flow streams of polyelectrolyte solutions [12]. Kantak et al. constructed micropillars to guide the oil droplets through parallel laminar streams consisting of two polymers and a washing solution [13]. By doing so, multiple layers of polymers were coated on the oil droplets. Song and Choi constructed slanted and suspended obstacles such that the smaller particles stay in the original stream and flow through the gap while the larger particles are diverted into the new stream [14]. Pan et al. utilized T-junction droplet microfluidic chips to perform particle coating where the dispersed phase (high-molecular-weight polyvinyl alcohol, H-PVA) containing polystyrene (PS) shells was sheared by the continuous phase (oil) to generate droplets. Coating was then achieved when the H-PVA was grafted to the surface of the PS shells [15], which has applications for gas retention in inertial confinement fusion experiments. In recent years, an acoustic radiant force has been applied to manipulate particles to achieve a conformal coating of microparticles and cells [16]. Compared to other strategies, acoustofluidics offers unique advantages such as contact-free and nondestructive sample manipulation, biocompatibility, no strict requirements on the composition of the working fluid, samples with free labels, and a wide range of particle size and properties [17]. Ayan et al. implemented tilted-angle standing surface acoustic waves (taSSAWs) into the microchannel, which has multiple inlets for the injection of different solutions [16]. The solutions formed laminar flow streams inside the microchannel, and the particles or cells were subjected to taSSAWs, such that they traversed from one side of the channel to the other side, and conformal coating was then achieved when the particles passed through the coating streams. In this study, we intended to apply traveling surface acoustic waves (TSAWs) to perform conformal coating by pushing the particles through laminar flow streams. For TSAWs, since only one interdigital transducer is used, less complication in chip design, feasibility in repetitive coating, and a smaller footprint of the microchip are expected. In addition, the magnitude of a TSAW is greater than that of a SSAW when the particle size is approximately larger than 8 μm [18].

## 2. Materials and Methods

### 2.1. Materials

The lithium niobate wafer (LiNbO_3_, 128°, Y-cut, X-propagation) was purchased from Ultimate Materials Technology, Co., Ping Tung, Taiwan. The polydimethylsiloxane (PDMS, Sylgard 184) was purchased from Dow Corning, Midland, MI, USA. The positive photoresist (S1818) was purchased from J. T. Baker, Phillipsburg, NJ, USA. The negative photoresist (SU-8 2025) was purchased from Gersteltec, Pully, Switzerland. The gold etchant was purchased form Thermo Fisher, Waltham, MA, USA. N-dodecane was purchased from Alfa Aesar, Ward Hill, MA, USA. Polyallylamine hydrochloride (MW: 17,000, 20%) and bovine serum albumin were purchased from Sigma-Aldrich, Burlington, MA, USA. The 10 μm fluorescent latex beads and the carboxylate conjugated polystyrene particles were purchased from Thermo Fisher, Waltham, MA, USA.

### 2.2. Fabrication of Acoustofluidic Assembly

**Figure 1:**
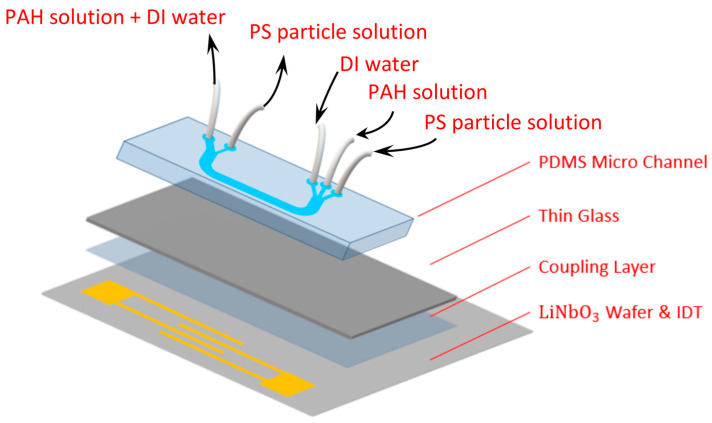
Schematics of acoustofluidic assembly.

Figure 1 shows the schematics of the acoustofluidic assembly for the experiments. It consists of three components, i.e., the acoustofluidic microchip, the coupling layer, and the interdigital transducer (IDT). For the acoustofluidic microchip, a U-shaped PDMS microchannel was used, where the width, depth, and length of the microchannel were 200 μm, 100 μm, and 4 cm, respectively. Due to the configuration of connecting the tubes to the microchip, the electrode to the functional generator and power supply, etc., the design of a U-shaped microchannel was used, which allows the interdigital electrode to be placed as close to the microchannel as possible with a smaller footprint. There were three inlets, where the PAH solution was the middle stream and the particle solution and DI water were the sheath streams. To collect the coated microparticles without or with minimum PAH in the collected solution, only two outlets were exploited, where the width of the channel for particle collection was smaller than that for the waste solution. The microchannel was constructed through soft lithography by first obtaining the photoresist channel structure on a silicon wafer. The mixture of the PDMS base and curing agent in a ratio of 10:1 was thoroughly stirred, degassed, and gently poured onto the silicon wafer, followed by placing it in a 65 °C oven for 4 h. The PDMS microchannel was then peeled off from the wafer, and three inlet and two outlet reservoirs with 1 mm diameters were drilled. The microchannel was subsequently subject to plasma treatment (PDC-001, Harrick, Pleasantville, NY, USA) for 5 min and bonded to a cleaned glass slide. As for the IDT, a 20 nm thick Ti layer and a 100 nm thick gold layer were successsively e-beam sputtered (FU-12 PEB, FSE Corporation, Hsinchu, Taiwan) on the cleaned LiNbO_3_ wafer. The patterned photoresist structure was obtained through standard photolithography. The substrate was then immersed in the gold etchant, where the gold layer was etched away, followed by immersing it in the solution of amonia and hydrogen peroxide at a ratio of 1:1 to remove the Ti layer. The photoresist was subsequently removed by rinsing with acetone, and a set of IDTs with 27 pairs wasobtained with a width, spacing, period, and aperture of 10 μm, 10 μm, 40 μm, and 5 mm, respectively. The wavelength of the SAW was 40 μm, corresponding to a resonance frequency of 99.75 MHz. For the coupling layer, it was applied in order to reuse the IDT, where the acoustofluidic microchip was simply discarded after usage, and the related study can be found in the literature [19]. In brief, a tiny droplet of n-dodecane was dispensed on the IDT substrate. The PDMS–glass superstrate was then placed on top of the droplet and slightly pressed such that the n-dodecane wicked through the interstitial space to form the coupling layer between the IDT substrate and the superstrate.

### 2.3. Conformal Coating of Microparticles

The polystyrene microparticles were intended to be coated with a positively charged polyelectrolyte, PAH. The PS microparticle solution, PAH solution, and DI water were separately injected into the microchannel by syringe pumps (Legato 100, KD Scientific, Holliston, MA, USA) from three inlet reservoirs, where the PAH solution was the middle stream. After the microchannel was filled with the solutions, the AC power was then turned on, where the surface acoustic wave was generated by a function generator (AFG3101, Tektronix, Beaverton, OR, USA) with an amplifier (A009K251-4444R, B&K, Marlborough, MA, USA). The upright microscope (Eclipse Ni, Nikon, Tokyo, Japan) with a monochrome CMOS sensor (DS-Qi2, Nikon, Tokyo, Japan) was used to minitor the movement of the microparticles. The PS microparticle solution was collected from one outlet, and the other two streams were collected from another outlet. Befoe the experiments, the microchannel was treated with 1% bovine serum albumin solution for 3 min to minimize particle adhesion to the microchannels.

### 2.4. Characterization of the Coated Microparticles

To assess the adsorption of the polyelectrolyte to the microparticles, the zeta potential of the coated microparticles was measured by using dynamic light scattering (DLS). In DLS, a laser beam is directed into the sample containing the microparticles, which undergo random Brownian motion in suspension. The light is scattered and, since the positions of the microparticles fluctuate due to Brownian motion, its intensity varies as the microparticles move. The information regarding time-dependent changes in light intensity is then obtained through a correlation function analysis, which allows the diffusion coefficient of the microparticles to be determined. The diffusion coefficient is subsequently related to the particle size and the viscosity of the solvent through the Stokes–Einstein equation. Finally, from the Smoluchowski equation, the zeta potential is calculated from the measured electrophoretic mobility, the dielectric constant, and the viscosity of the medium. Hence, the zeta potential of the uncoated and coated microparticles was measured through dynamic light scattering (Zetasizer Nano-Zs, Malvern Instruments, UK). For comparison, the purchased microparticles were first centrifuged, followed by removing the supernatant. The PAH solution was added in the vial with sediment, which was thorouly mixed. The solution was then centrifuged again, followed by removing the supernatant, adding DI water, and mixing for subsequent analysis.

## 3. Results and Discussion

### 3.1. Laminar Flow Inside the Microchannel

**Figure 2:**
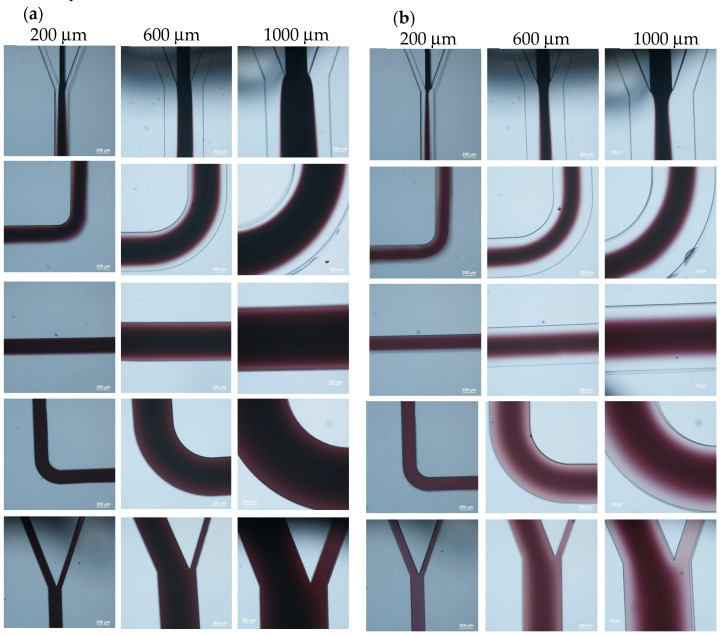
Flow streams inside the microchannel. The flow ratio between the center stream and sheath stream is (**a**) 1:1 and (**b**) 1:6.

In order to realize the coating of the microparticles, the first step is to ensure a laminar flow inside the microchannel such that the entire microchannel will not be filled with PAH. To examine the flow streams inside the microchannel, the PAH stream was replaced with ink solution, and Figure 2 shows the flow pattern inside the microchannels with three different widths. The flow rate was 0.08 mL/h for each stream inside the 200 μm-wide microchannel, 0.24 mL/h for each stream inside the 600 μm-wide microchannel, and 0.4 mL/h for each stream inside the 1000 μm-wide microchannel. It can be seen that, for the 200 μm-wide microchannel, a laminar flow was observed only in the inlet section of the microchannel, as shown in Figure 2a. At the first turn of the microchannel, the ink diffused across the channel and occupied the entire microchannel as it further proceeded to the outlet. As the width of the microchannel increased to 600 μm, laminar flow was observed up to the first turn of the microchannel. Although there was a color difference between the middle stream and the sheath streams in the middle section of the microchannel, the mixing caused by diffusion could not be neglected, and the laminar flow patterns could not be recognized afterwards. A similar phenomenon was observed for the microchannel that was 1000 μm in width. To prevent the middle stream from completely mixing with the sheath flows through diffusion, the flow rate of the middle stream was decreased to 1/6 of that of the sheath streams. That is, the width of the sheath flows was increased, and the flow pattern is shown in Figure 2b. For the 200 μm-wide microchannel, the flow pattern was similar to that in the previous case. As the width of microchannel increased to 600 μm, a clear laminar flow pattern was observed up to the middle section of the microchannel. Although there was a light color observed in the sheath flow streams, the mixing caused by diffusion was not severe. Moreover, only the particle solution was directed to the right reservoir, ensuring no contamination of PAH after particle collection. For the 1000 μm-wide microchannel, a laminar flow pattern was observed throughout the entire channel. Hence, a flow rate ratio of 1:1/6:1 for the particle stream, PAH stream, and DI water stream was applied for the subsequent experiments. Moreover, since there was no laminar flow pattern in the 200 μm-wide microchannel, it was not used for the coating of microparticles.

### 3.2. Movement of Microparticles under Traveling Surface Acoustic Wave

**Figure 3:**
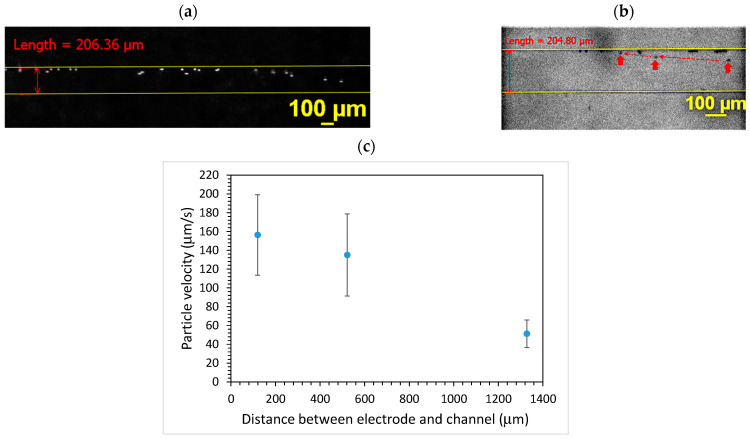
The particle movement under TSAWs inside the microchannel. (**a**) PS particles and (**b**) carboxylate-conjugated PS particles. (**c**) Effect of distance between electrode and microchannel on particle moving speed.

Figure 3 shows the movement of microparticles under the effect of TSAWs. It can be seen that the particles moved from one side to the other side of the 200 μm-wide microchannel after turning on the AC. The particles took approximately 0.8 s to cross the microchannel. Since the average flow speed was approximately 2.4 mm/s, this leads to a 1.92 mm travel distance in the axial direction, which is well within the effect of TSAWs. For the 1000 μm-wide microchannel, the particles took approximately 2.4 s to cross the microchannel. With approximately the same average flow speed (2.4 mm/s), the travel distance in the axial direction was 5.76 mm and still within the effect of TSAWs. The effect of the distance between the electrode and the microchannel was examined, as shown in Figure 3c. The distance was measured between the edge of the microchannel and the edge of the electrode under the microscope. It can be seen that, as the distance between the electrode and the microchannel increased, the particle moving speed decreased. This is due to the attenuation of the surface radiant force when the surface acoustic wave travels a longer distance. Therefore, the edge of the electrode was placed under the microscope and adjusted as close to the edge of the microchannel as possible, and the time taken for the microparticles to move across 200, 600, and 1000 μm was approximately 1.3, 3.8 and 6.3 s, respectively.

### 3.3. Coating of Microparticles

#### 3.3.1. Unfunctionalized PS Particles

**Figure 4:**
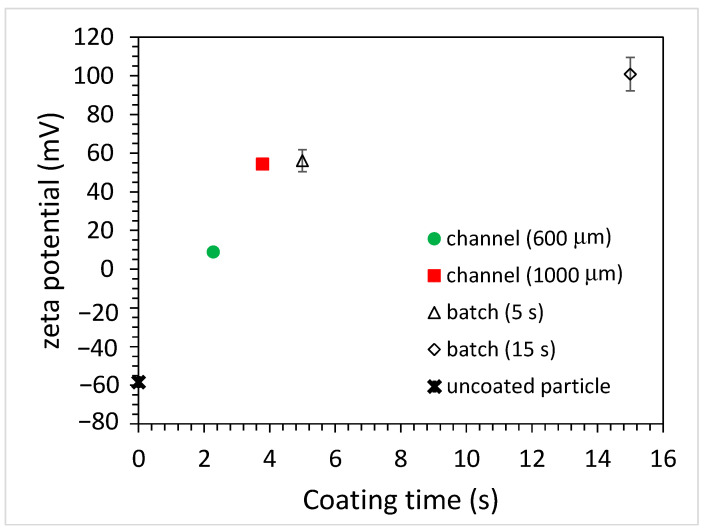
The zeta potential of coated PS particles for batch and microfluidic approaches (PAH concentration: 10 mg/mL).

**Figure 5:**
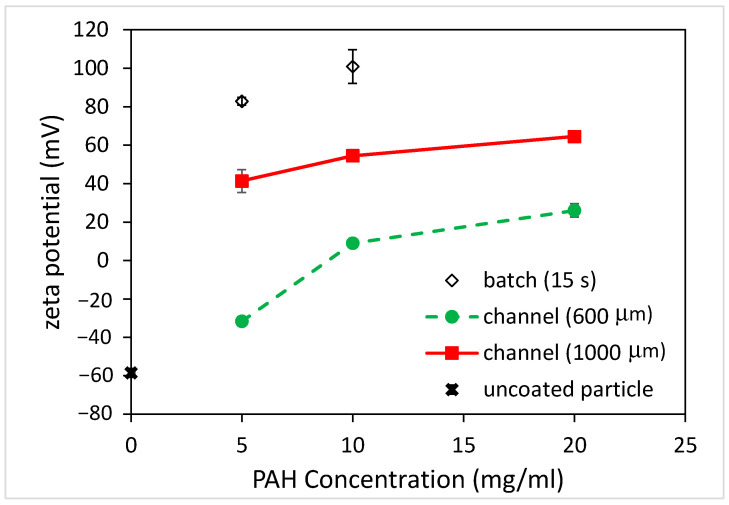
The effect of PAH concentrations on zeta potential of coated PS particles for batch and microfluidic approaches (coating time for batch: 15 s).

When polyelectrolytes (PEs) are dissolved in a colloidal particle suspension, they undergo irreversible adsorption onto the particle surfaces. If the overall PE concentration is low, the PEs will adsorb onto the particle surfaces until no PE remains in the solution. The particle surfaces are partially covered with PEs, which is referred to as an unsaturated layer [20]. As the concentration of the PEs increases, the number of adsorbed PEs also increases, to the point where no more PEs can adsorb onto the particle surfaces. At this concentration and beyond, a saturated layer forms and the excess PEs will remain in the solution. This adsorption behavior will influence the particle charge, which in turn affects the zeta potential of the coated particles. One of the defining features is the occurrence of a charge reversal, commonly known as overcharging [20]. That is, the negative (or positive) zeta potential of the particles is decreased as the adsorbed amount of positively (or negatively) charged PEs onto the particle surface increases. As the concentration of PEs exceeds the critical adsorption concentration, the charge reversal occurs and the zeta potential changes from negative to positive or vice versa. By further increasing the PE concentration beyond a certain value, there will be a saturation effect where the PE concentration does not significantly impact the zeta potential. Figure 4 shows the zeta potential of the coated PS microparticles using the batch and microfluidic approaches. For the unfunctionalized PS particles, the zeta potential was measured to be negative (approximately −60 mV). After immersing the particles in 10 mg/mL of PAH solution for 5 s, a charge reversal occurred and the zeta potential became positive (approximately +60 mV), indicating that the surface of the PS particles was coated with PAH. By further increasing the immersion time to 15 s, the zeta potential increased to be approximately +100 mV. As for coating microparticles using the microfluidic approach, the zeta potential of microparticles altered when using microchannels with different widths. When using the 600 μm-wide microchannel, the zeta potential became positive (approximately +10 mV, charge reversal) and increased up to approximately +60 mV as the channel width increased to 1000 μm. It was rationalized that, as the channel width increased, the width of the middle stream (PAH solution) increased as well and the duration for the microparticles traversing the middle stream became longer. Since the time taken for the microparticles to traverse the 600 and 1000 μm-wide microchannels was 3.8 and 6.3 s, respectively, the estimated time taken for them to cross the middle stream was around 0.29 and 0.48 s due to the flow rate ratio of 1 to 6 between the middle stream and the sheath stream. However, from Figure 2b, the actual width of the middle stream was approximately three times wider compared to that of the sheath flow in the middle section of the microchannel owing to the diffusion effect. Hence, the times were re-estimated to be 2.28 and 3.78 s. Note that the zeta potential of the coated microparticles when using the 1000 μm-wide microchannel was similar to that obtained when using the batch method with a 5 s immersion time. Another interesting finding is that the error bar of the zeta potentials of the coated microparticles was much smaller when adopting the microfluidics approach. This implies that the coating of the individual microparticles was more uniform as they passed through the PAH stream, which might result from the nature of the laminar flow of the coating stream. The concentration of PAH solution will also influence the zeta potential of the coated microparticles, as shown in Figure 5.

For the batch method, the zeta potential of the coated microparticles was approximately +80 mV when using the 5 mg/mL PAH solution with a 15 s immersion time and increased to approximately +100 mV when using the 10 mg/mL PAH solution. As for the microfluidics approach, the zeta potentials all became more “positive” when using a higher PAH concentration for all channels with different widths. For the 600 μm-wide microchannel, the negative zeta potential of microparticles decreased (from −60 to −30 mV) when using the 5 mg/mL PAH solution. The charge reversal occurred (from −30 to +10 mV) when a 10 mg/mL PAH concentration was used. As the PAH concentration increased to 20 mg/mL, the zeta potential further increased to +30 mV. For the 1000 μm-wide microchannel, the charge reversal occurred (from −60 to +40 mV) when using the 5 mg/mL PAH solution. The zeta potential became +55 and +65 mV when using the 10 and 20 mg/mL PAH concentrations, respectively.

The measurement of zeta potential obtained utilizing dynamic light scattering (DLS) can be used to assess the stability of colloidal suspensions. When particles are coated with polyelectrolytes, the resulting changes in surface charge can affect the zeta potential. A high absolute zeta potential value (either positive or negative) indicates strong electrostatic repulsion between particles, contributing to colloidal stability. Conversely, a low zeta potential may lead to particle aggregation. When the magnitude of the zeta potential (mV) is between 0 and 5, rapid coagulation or flocculation occurs [21]. When it is between 10 and 30, the colloids are incipiently instable. As its value becomes larger than 30, the colloids are stable in the suspension, where three levels of stability are categorized. That is, 30 to 40 refers to moderate stability, 40 to 60 refers to good stability, and larger than 60 refers to excellent stability. Therefore, the batch method yielded a stable colloidal suspension at the 5 and 10 mg/mL PAH concentrations. For the microfluidics approach, the coated microparticles were not stable when using the 600 μm-wide microchannel as its zeta potentials were between −30 and +30 mV. On the other hand, a stable colloidal suspension can be obtained by using a 1000 μm-wide microchannel. This indicates that, just like the batch method, the microfluidic approach can be exploited to coat the microparticles with polyelectrolytes and yield a stable colloidal suspension.

#### 3.3.2. Carboxylate-Conjugated PS Microparticles

**Figure 6:**
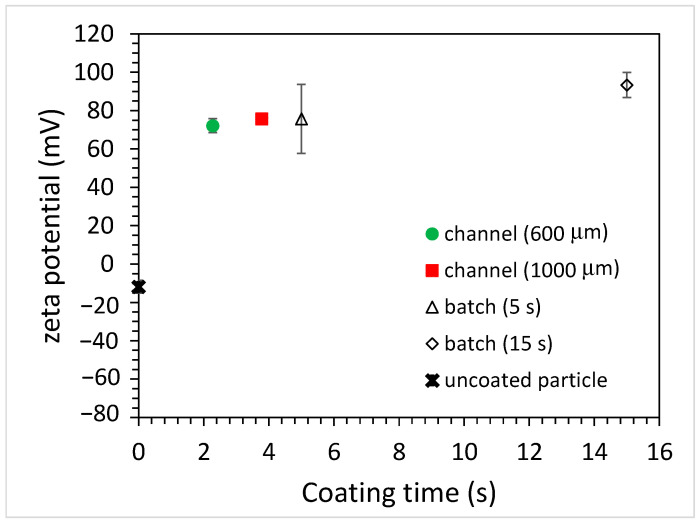
The zeta potential of coated, carboxylate-conjugated PS microparticles for batch and microfluidic approaches (PAH concentration: 10 mg/mL).

**Figure 7:**
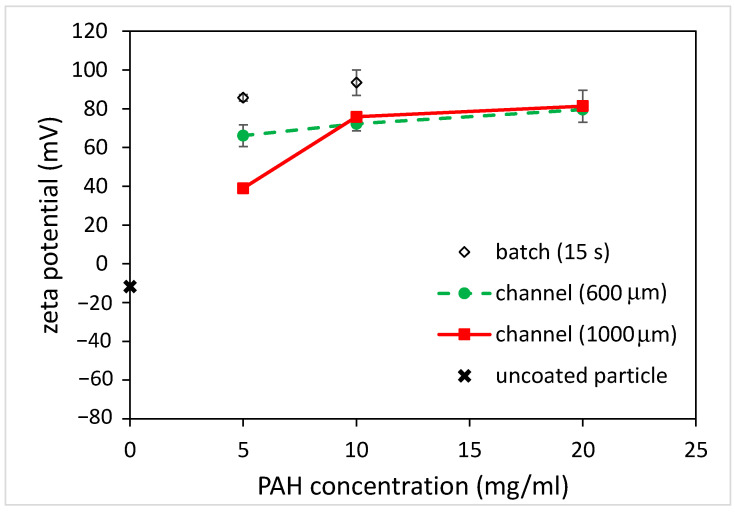
The effect of PAH concentrations on zeta potential of coated, carboxylate-conjugated PS particles for batch and microfluidics approaches (coating time for batch: 15 s).

As for the carboxylate-conjugated PS microparticles, the zeta potential was measured to be slightly negative (approximately −10 mV), and it became positive (approximately +75 mV) after being immersed in the PAH solution for 5 s, as shown in Figure 6. This indicates that the surfaces of the carboxylate-conjugated PS microparticles were successfully coated with PAH. When the immersion time increased to 15 s, the zeta potential was approximately +90 mV. Although this increase in the zeta potential was observed due to a longer immersion time, the amount of increase in the zeta potential (15 mV) was less compared to that (40 mV) for the unfunctionalized PS particles. It was hypothesized that negatively functionalized PS particles could be beneficial for attracting positively charged PAH. As for coating microparticles using the microfluidics approach, the zeta potential of the microparticles again changed when using the microchannels with different widths, as shown in Figure 6. For the 600 μm-wide microchannel, a charge reversal occurred and the zeta potential was approximately +72 mV. When using the 1000 μm-wide microchannel, the zeta potential slightly increased to approximately +76 mV. Since the channel width increased, the width of the middle stream (PAH solution) increased, and the time taken for the microparticles to traverse the middle stream increased. However, unlike the unfunctionalized particles, for which the magnitude of the zeta potential depends on the channel width, the zeta potentials of the coated particles all changed from negative to positive, and the magnitudes were similar to each other. Moreover, the magnitude was similar to that obtained when using the batch method with a 5 s immersion time. This implies that the coating of microparticles can be achieved in a shorter time using the microfluidic approach. Figure 7 shows the effect of PAH solution concentrations on the zeta potential of the coated microparticles. For the batch method, it can be seen that the zeta potential of the coated microparticles was approximately +86 mV when using the 5 mg/mL PAH solution with a 15 s immersion time, which is comparable to that obtained (+93 mV) when using the 10 mg/mL PAH solution. As for the microfluidic approach, the zeta potentials were all increased when using a higher PAH concentration for all the channels with different widths. Although there was a discrepancy in the measurement of the zeta potential when using a 5 mg/mL PAH solution, which might be due to a potential aggregation of PAH at a solution pH between 5 and 7 [22], the results were comparable, and the effect of the negative functional group on particle surface coating was conspicuous. The finding also shows that, at the same PAH concentration, the effect of microchannel width on the measured zeta potential was not substantial compared to that when using the unfunctionalized particles. For functionalized particles, the microfluidic approach can yield a stable colloidal suspension.

## 4. Conclusions

In this paper, the conformal coating of microparticles was demonstrated by using a traveling-surface-acoustic-wave-based microchip. Under the effect of TSAWs, the microparticles moved from one side of microchannel to the other side, where they traversed the coating stream and were conformally coated. For the unfunctionalized PS microparticles, the zeta potential of the coated microparticles increased as the microchannel width and concentration of PAH solution increased. As for the carboxylate-conjugated PS microparticles, the zeta potentials of the coated microparticles all became positive, and their magnitudes were similar to each other despite utilizing different channel widths and PAH concentrations. Thus, the coating of functionalized particles could be achieved in a shorter time. In addition, since the coating time can be adjusted by using different channel widths (or flow rate ratios), the microfluidic approach is potentially useful for assessing the polyelectrolyte adsorption onto the particles in a shorter time (less than 5 s) that would be difficult to achieve when using the batch method.

## Data Availability

Not applicable.
